# The complete chloroplast genome of the marine microalgae *Chaetoceros muellerii* (Chaetoceroceae)

**DOI:** 10.1080/23802359.2020.1869608

**Published:** 2021-02-08

**Authors:** Yajun Li, Xiaodong Deng

**Affiliations:** Hainan Provincial Key Laboratory for Functional Components Research and Utilization of Marine Bio-resources, Institute of Tropical Bioscience and Biotechnology, Hainan Academy of Tropical Agricultural Resource, Chinese Academy of Tropical Agricultural Sciences, Haikou, P. R. China

**Keywords:** *Chaetoceros muellerii*, chloroplast genome, phylogenetic analysis, marine microalgae

## Abstract

In this study, the complete chloroplast genome of *Chaetoceros muellerii* was sequenced by using PacBio sequencing platform. The chloroplast genome was 116,284 bp in length, harboring a large single copy (LSC) region of 61,946 bp, a small single copy (SSC) region of 39,308 bp, and a pair of inverted repeats (IR) regions of 7515 bp. The overall GC content was 30.86%. The chloroplast genome of *C. muellerii* contained 131 genes in total, including 95 protein-coding genes, 30 transfer RNA (tRNA) genes and 6 ribosomal RNA (rRNA) genes. The phylogenetic analysis revealed that *C. muellerii* was closely related to *Chaetoceros simplex* with 100% bootstrap values. This study will contribute to the phylogenetic and taxonomic analysis of species in the family Chaetoceroceae.

Chaetoceros Ehrenberg 1844 is the unique genus in the Chaetoceroceae (Biddulphiales, Bacillariophyta) with more than 500 recorded species and infraspecific taxa (Guiry and Guiry 2020). As the largest genus of planktonic diatom, Chaetoceros species are wildly distributed from polar to tropical waters, and play an important role in global carbon cycle and aquatic ecosystems (Nelson et al. 1995). As reported, *Chaetoceros muellerii* contains the fruitful lipids and has the nutrition value for the growth requirements (Liang et al. 2020). To data, the growth requirements, nutrition value and lipid extraction of *C. muellerii* have been well documented (Naghdi et al. 2016; Kumaran et al. 2017; Remize et al. 2020). However, little information is available about genetic and genomic researches on *C. muellerii*. In this study, the complete chloroplast genome of *C. muellerii* was sequenced and assembled by using the third-generation sequencing technology, the genome sequence has been submitted to NCBI GenBank with an accession number of MW004650.

*C. muellerii* strain was obtained from Center for Collections of Marine Algae of Xiamen University (CCMA-187, N24.61°, E118.32°). The Chloroplast DNA was isolated with Plant Chloroplast DNA column extraction kit (BioRab, Beijing) according to the instructions of the manufacturer, and sequenced by combining Illumina Hiseq4000 and PacBio sequencing platform at Nextomics Biosciences Co. Ltd (Wuhan, China). PacBio library construction was mainly based on the PacBio Sample Net-Shared Protocol (https://www.pacb.com/wp-content/uploads/2015/09/Shared-Protocol-10-kb-to-20-Kb-Template-Preparation-with-Low-Input-DNA.pdf). Approximately 200 ng of cpDNA were sheared to 15–20 kb via a covaries g-tube device, and then the DNA fragments were end-repaired and ligated SMRTbell adapters. After annealing sequencing primer and binding polymerase to SMRTbell templates, the PacBio library was constructed and sequenced on the PacBio Sequel system, yielding a total of 7924.1 Mb of subreads. For Illumina Hiseq sequencing, approximately 1 μg cpDNA was sheared to 300–500 bp using Covaris M220 and the sequencing libraries were constructed using TruSeq™ Nano DNA Sample Prep Kit. After 8-cycle PCR amplification, purification, quantification and validation, the DNA libraries were sequenced on Illumina Hiseq system. Total 7384.5 Mb of raw data was generated and then filtered using Trimmomatic 0.39 (Bolger et al. 2014), obtaining 6530.1 Mb of clean data. The resulting clean data of Illumina Hiseq and the PacBio data were used for De novo assembly of the chloroplast genome using NOVOPlasty v2.7.2 (Dierckxsens et al. 2016) with *Saccharum hildebrandtii* (GenBank: MF563371.1) as the reference. GapCloser V1.12 software (Luo et al. 2012) was used to perform vulnerability completion and base correction. The chloroplast genome annotation was conducted by online software GeSeq (Tillich et al. 2017).

The chloroplast genome length of *C. muellerii* was 116,284 bp with a typical quadripartite structure, which compose of 61,946 bp of a large single copy (LSC) region, 39,308 bp of a small single copy (SSC) region and 7515 bp of two inverted repeats (IR) regions. The overall GC content is 30.86%. A total of 131 genes (130 unique genes) were predicted in the chloroplast genome of *C. muellerii*, including 95 protein-coding genes, 30 transfer RNA genes and 6 ribosomal RNA genes. Moreover, none of gene contains intron. Three rRNAs (rns, rnl and rrn5) and three tRNAs (trnp-UGG, trnl-GAU and trnA-UGC) were located in the repeats regions. The IR regions also contain photosystem II protein Y (psbY), acyl carrier protein (acpP) and hypothetical chloroplast reading frames 89(ycf89). Ribosomal protein 32 (rpL32) was located in the border of IRA and SSC, and ycf45 was located in the border of IRA and LSC.

Phylogenetic analyses were performed using maximum likelihood (ML) in PhyloSuite using the concatenated coding sequences of 116 chloroplast coding genes for 37 species of Bacillariophyta (Zhang et al. 2020). Supports for nodes were calculated via 5000 ultrafast bootstrap replicates. The result of phylogenetic analysis revealed that *C. muellerii* was suggested more closely to *Chaetoceros simplex*, forming a clade with *Acanthoceras zachariasii* and *Cerataulina daemon* in this study (Figure 1).

**Figure 1.**
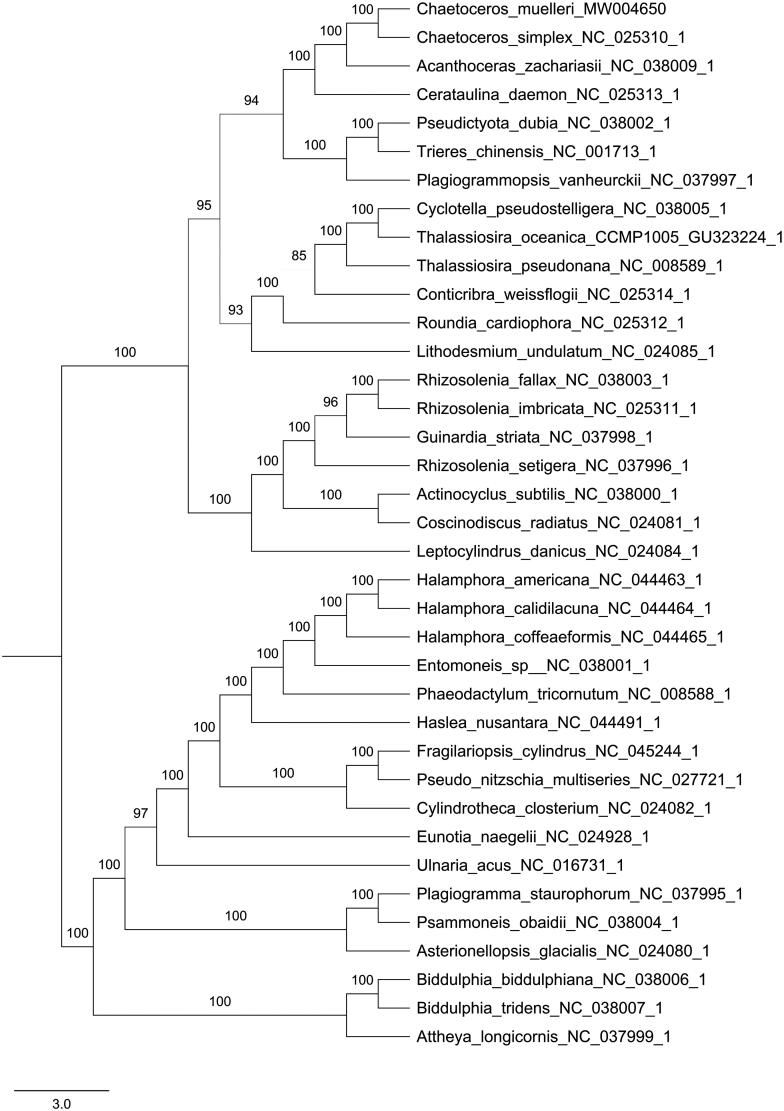
Phylogenetic relationships of 37 species based on concatenated coding sequences of 116 chloroplast coding genes. The phylogenetic analysis was performed by using the software PhyloSuite. The sequences were aligned by MAFFT v7.037 and concatenated, and then the data was partitioned using PartitionFinder2 with AICc model selection under GTR, GTR + G and GTR + I+G + X models. The IQ-tree was used to infer the maximum likelihood (ML) tree with 5000 ultrafast bootstraps under Partition Mode.

In conclusion, the complete chloroplast genome sequence of *C. muellerii* can provide a reliable barcode for understanding the phylogeny and evolution of Chaetoceroceae species. In the next study, we will analyses the conserved and variable regions of *C. muellerii* cpDNA sequence, and develop effective DNA barcodes to accurate identification of Chaetoceroceae species.

## Data Availability

The genome sequence data that support the findings of this study are openly available in GenBank of NCBI at (https://www.ncbi.nlm.nih.gov/) under the accession no. MW004650. The associated BioProject, SRA, and Bio-Sample numbers are PRJNA684148, SRS7869517, and SAMN17052596 respectively.
